# *Acanthopagrus oconnorae*, a new species of seabream (Sparidae) from the Red Sea

**DOI:** 10.1111/jfb.15147

**Published:** 2022-08-05

**Authors:** Lucía Pombo‐Ayora, Viktor N. Peinemann, Collin T. Williams, Song He, Yu Jia Lin, Yukio Iwatsuki, Donal D. C. Bradley, Michael L. Berumen

**Affiliations:** ^1^ Red Sea Research Center, Division of Biological and Environmental Science and Engineering King Abdullah University of Science and Technology Thuwal Saudi Arabia; ^2^ Center for Environment and Water, Research Institute King Fahd University of Petroleum and Minerals Dhahran Saudi Arabia; ^3^ Department of Marine Biology and Environmental Sciences, Faculty of Agriculture University of Miyazaki Miyazaki Japan; ^4^ Division of Physical Science and Engineering King Abdullah University of Science and Technology Thuwal Saudi Arabia

**Keywords:** biodiversity, new species, phylogeny, Red Sea, seabream, Sparidae, taxonomy

## Abstract

A new species of sparid fish, *Acanthopagrus oconnorae*, is described based on 11 specimens collected in the shallow (0–1 m depth) mangrove‐adjacent sandflats of Thuwal, Saudi Arabia. The new species is distinguished from its congeners by the following combination of characters: second anal‐fin spine 12.8%–16.6% of standard length (SL); 3½ scale rows between the fifth dorsal‐fin spine and lateral line; suborbital width 5.7%–6.7% of SL; eyes positioned at the anterior edge of the head, often forming a weakly convex break in an otherwise gently curved head profile, when viewed laterally; caudal fin light yellow with black posterior margin (approximately half of fin); anal fin dusky grey, with posterior one‐fifth of the fin light yellow; black streaks on inter‐radial membranes of anal fin absent. The most similar species to *A. oconnorae* is *Acanthopagrus vagus*, which differs by the presence of a w‐shaped anterior edge of the scaled predorsal area, a more acute snout and black streaks on the inter‐radial membranes of the anal fin. Phylogenetic placement and species delimitation of *A. oconnorae* are discussed based on COI, CytB and 16S sequences. It is hypothesized that ecology and behaviour explain how this species avoided detection despite its likely occurrence in coastal areas of the Red Sea with historically high fishing pressure.

## 1 INTRODUCTION

*Acanthopagrus* Peters, 1855 (family Sparidae), is an Indo‐West Pacific fish genus characterized by the presence of strong dorsal‐fin spines appearing alternately broad and narrow on each side and an outer series of canine teeth, followed by molars in the inner region (Bauchot & Smith, 1984). They generally inhabit shallow, warm coastal waters and estuaries, ranging from the Middle East (the Red Sea and the Arabian Gulf) and south‐eastern Africa through South Asia to the Sea of Japan, Australia and New Caledonia (Hindell *et al*., 2008; Iwatsuki, 2013; Oosthuizen *et al*., 2016; Platell *et al*., 2007; Wallace, 1975). Currently, 20 species are recognized under the genus *Acanthopagrus*, 11 of which have been described or resurrected in the past two decades (Hasan *et al*., 2020; Iwatsuki & Carpenter, 2006, 2009; Iwatsuki *et al*., 2006, 2010; Iwatsuki & Heemstra, 2010, 2011; Iwatsuki, 2013).

The Western Indian Ocean region, including the Red Sea and the Arabian Gulf, represents eight species of the genus *Acanthopagrus*: *Acanthopagrus arabicus* Iwatsuki, 2013, *Acanthopagrus berda (Fabricius in Niebuhr, 1775)*, *Acanthopagrus bifasciatus (Fabricius in Niebuhr, 1775)*, *Acanthopagrus catenula (Lacepède 1801)*, *Acanthopagrus omanensis* Iwatsuki & Heemstra, 2010, *Acanthopagrus randalli* Iwatsuki & Carpenter, 2009, *Acanthopagrus sheim* Iwatsuki, 2013 and *Acanthopagrus vagus* (Peters 1855) (Eagderi *et al*., 2019; Golani & Fricke, 2018; Iwatsuki, 2013; Iwatsuki & Heemstra, 2010; Iwatsuki & Heemstra, 2011). Of these, three species are known to have more or less yellow or yellowish pelvic‐fin and anal‐fin colouration: *A. arabicus*, *A. sheim* and *A. vagus*. Nonetheless, along with other characters, they largely differ in the number of scale rows between the fifth dorsal‐fin base and the lateral line and/or on the occurrence of black streaks in the inter‐radial membranes between yellow anal‐fin rays (Iwatsuki, 2013; Iwatsuki & Heemstra, 2010).

Recent field investigations on the Red Sea coast of Thuwal, Saudi Arabia, yielded several specimens of *Acanthopagrus* with yellowish‐fin colouration. Nonetheless, after detailed morphological and genetic comparisons with other similar species, the authors conclude that these specimens represent a previously undescribed species. Here the authors describe *Acanthopagrus oconnorae* sp. nov. based on 11 specimens collected from Thuwal, Saudi Arabia.

## 2 MATERIALS AND METHODS

### 2.1 Specimen collection and morphological data

**FIGURE 1:**
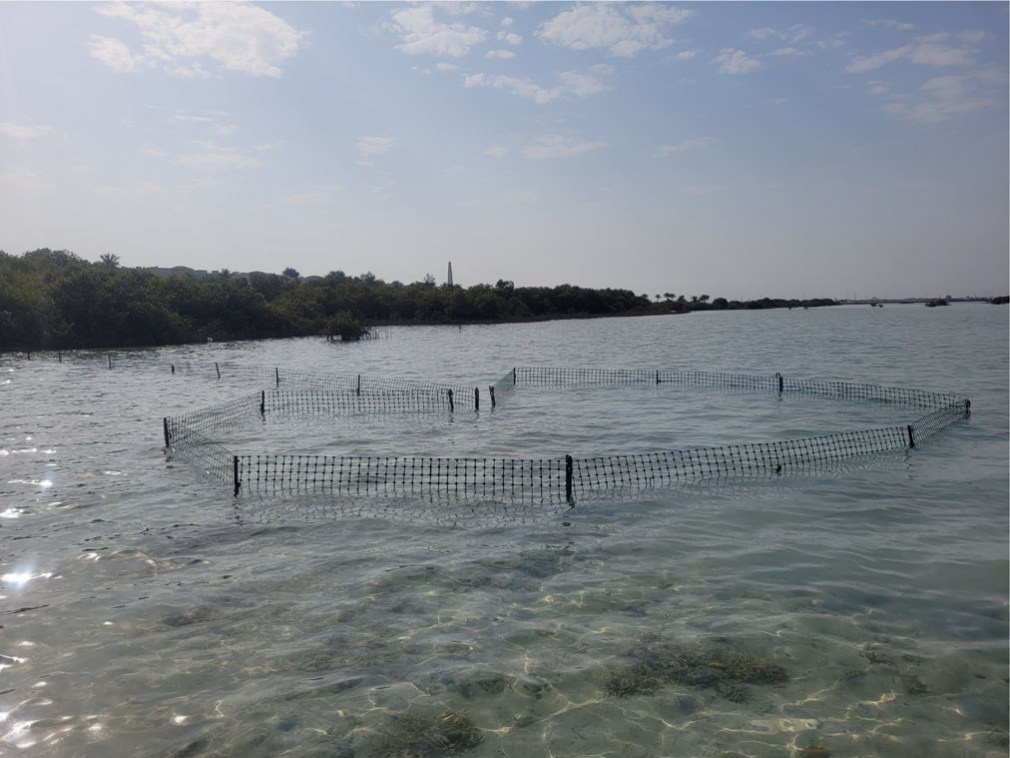
A tidal fish trap was used to capture specimens of *Acanthopagrus oconnorae*, near Thuwal in the central Saudi Arabian Red Sea. The trap was positioned over sandflat shelves with very shallow water (*c*. 1 m depth) near coastal stands of mangroves (*Avicennia marina*)

The authors collected 11 specimens of *A. oconnorae* sp. nov in shallow water (maximum 1 m depth at high tide) immediately adjacent to the mangroves near Thuwal in the central Red Sea (22.308367° N, 39.091016° E) (Figure 4). The first three specimens were collected using conventional hook‐and‐line gear, and the remaining eight specimens were captured using a tidal trap (Figure 1). A fin clip of each specimen was preserved in 96% ethanol for further molecular analysis. All morphologically analysed specimens were photographed alive, anaesthetized with lethal doses of MS‐222 (following Popovic *et al*., 2012) and then transported to the laboratory for further detailed photographs and analysis. All counts and measurements were made following Hubbs and Lagler (1964) with modifications from Iwatsuki *et al*. (2006) using a digital calliper with an accuracy of 0.1 mm. The dorsal‐most scales below the fifth and first dorsal‐fin spines, respectively, were counted as a half scale. Counts and measurements were taken on the left side of the body wherever possible. The calculated value for the second anal‐fin spine length/third anal‐fin spine length was abbreviated as 2AS/3AS. The authors then compared the meristics and morphometrics of their specimens to all nominal species of *Acanthopagrus* (Hasan *et al*., 2020; Iwatsuki, 2013).

### 2.2 Material examined in addition to type specimens

For a comparative morphometric and phylogenetic investigation among *Acanthopagrus* species, specimens of several other Western Indian Ocean representatives of *Acanthopagrus* spp. and *Rhabdosargus haffara* and *Sparidentex hasta* were obtained (see Supporting Information Table S1). One specimen of *A. berda* and five specimens of *R. haffara* (Fabricius in Niebuhr, 1775) were captured using the same tidal trap mentioned earlier. Individuals of *R. haffara* were released after a photograph was taken and a fin clip was collected. Specimens of *A. arabicus*, *A. bifasciatus*, *A. catenula* and *A. sheim* were obtained from a fish market in Dammam, on the eastern coast of Saudi Arabia. All specimens from the Damman fish market were caught by local fishermen in the Arabian Gulf except for *A. catenula*, which was shipped to the Dammam fish market from Oman.

Initial morphological identification of all specimens collected from Dammam was performed by Y.J.L. Whole specimens from the Dammam fish market were further analysed in the Reef Ecology Laboratory at the King Abdullah University of Science and Technology (KAUST) by V.N.P. and L.P.‐A. The morphological identification of these species was further confirmed by barcoding of COI and 16S fragments against available sequences on GenBank, including the hologenetypes of *A. sheim* and *A. arabicus* (Iwatsuki, 2013). The authors did not physically examine any specimen previously deposited in any ichthyological collection. Comparisons to deposited specimens were made only *via* published meristic and morphometric measurements.

Collection sample details of the comparative materials, including the number of specimens used to calculate the K2P distances, are provided as follows: number of specimens, collection method, main basin, country, locality and GenBank accession numbers. Specimens of *A. catenula* were imported to the Damman fish market from Oman, but the authors do not have more specific (*e.g*., basin or locality) information; *R. haffara* (five specimens, tidal trap, Red Sea, Saudi Arabia, Thuwal, COI: OM811744‐48, 16S: OM831076‐77, CytB: OM830883); *R. haffara* (eight specimens, fish market, Arabian Gulf, Saudi Arabia, Dammam, COI: OM811734‐43, 16S: OM831065‐71, CytB: OM830875‐82); *A. berda* (three specimens, tidal trap, Red Sea, Saudi Arabia, Thuwal, COI: OM811723‐25, 16S: OM831055‐57, CytB: OM830842‐43); *A. arabicus* (eight specimens, fish market, Arabian Gulf, Saudi Arabia, Dammam, COI: OM811686‐93, 16S: OM831013‐19, CytB: OM830838‐41); *A. sheim* (15 specimens, fish market, Arabian Gulf, Saudi Arabia, Dammam, COI: OM811696‐710, 16S: OM830852‐64, CytB: OM831028‐1042); *A. bifasciatus* (four specimens, fish market, Arabian Gulf, Saudi Arabia, Dammam, COI: OM811726‐29, 16S: OM831058‐64, CytB: OM830844‐47); *A. catenula* (four specimens, fish market, Oman, COI: OM811730‐33, 16S: OM831059‐61, CytB: OM830848‐51); and *S. hasta* (two specimens, fish market, Arabian Gulf, Saudi Arabia, Dammam, COI: OM811694‐95, 16S: OM831020‐22, CytB: OM830885‐87).

### 2.3 Molecular data, phylogenetic analysis and DNA‐based species delimitation

The authors used the Qiagen Blood and Tissue Kit with 56°C overnight incubation for all DNA extractions. The mitochondrial fragments of the regions COI, CytB and 16S were amplified. COI was amplified using the universal fish primers FishF1 and FishR2 (Ward *et al*., 2005), CytB using the primers Cyb9 (Song *et al*., 1998) and Cyb7 (Taberlet *et al*., 1992) and 16S using the primers 16SF and 16SR (Palumbi, 1996). The thermal profiles for COI, CytB and 16S were as follows: 15 min of denaturation at 95°C, followed by 35 cycles of 30 s of denaturation at 94°C, primer annealing for 1 min at 50°C and 1 min extension at 72°C, with a final extension of 10 min at 72°C.

Published partial sequences of COI, CytB and 16S of all available fishes of the genus *Acanthopagrus* were downloaded from GenBank (Sayers *et al*., 2011) to build a phylogeny and position *A. oconnorae* in a phylogenetic context. Details of the accession numbers of the sequences along with supporting data are provided in Supporting Information Table S1. All sequences were edited and concatenated into alignments using the alignment function based on the MAFFT algorithm (Kazutaka *et al*., 2009) in GENEIOUS 2021.2 (http://www.geneious.com; Kearse *et al*., 2012). Some of the species of this study are missing at least one of the fragments (COI, 16S or CytB) in public repositories. Phylogenetic analysis was performed with concatenated partial sequences of COI, CytB and 16S using maximum likelihood on the online platform for IQtree (Trifinopoulos *et al*., 2016). This approach allows us to include samples missing one locus, thus maximizing the number of samples and species in the phylogenetic analysis. The branch support was analysed with SH‐aLRT (Anisimova *et al*., 2011), aBayes support (Guindon *et al*., 2010) and ultrafast bootstrap (Minh *et al*., 2013).

For DNA‐based species delimitation, the authors used two methods based on distance matrices of the COI alignments and one method based on the COI phylogenetic tree. For the first two approaches, they used genetic distance matrices based on the COI alignments using the Kimura 2‐parameter as inputs for the Assemble Species by Automatic Partition (ASAP, Puillandre *et al*., 2021) and the distance‐based Automatic Barcode Gap Discovery (ABGD, Puillandre *et al*., 2012). ABGD was conducted at https://bioinfo.mnhn.fr/abi/public/abgd/abgdweb.htm using the following parameters: Pmin = 0.001, Pmax = 0.1, steps = 100, X = 1.5 and Nb bins = 100. ASAP species delimitations were analysed at https://bioinfo.mnhn.fr/abi/public/asap/asapweb.html considering only the species partition models with the lowest ASAP score. For the tree‐based method, the authors used a Bayesian framework of the multi‐rate Poisson tree process (mPTP, Kapli *et al*., 2017) at https://mptp.h-its.org/#/tree. In addition, K2P divergence distances between the *Acanthopagrus* species present in the Western Indian Ocean were calculated for COI and 16S fragments.

### 2.4 Ethics statement

The collection of live individuals in this study was carried out under the ethics protocol numbers 18IACUC14 and 19IACUC03 issued by the KAUST Institutional Animal Care and Use Committee. The exception is the first individual which was caught in the course of recreational fishing. All subsequent live individuals targeted for this study followed the ethics protocols described in Section 2.1.

### 2.5 Nomenclatural acts

The species names presented in this article comply with the International Code of Zoological Nomenclature (ICZN). The new names contained in this article have been registered in ZooBank, the electronic registration system for the ICZN. The ZooBank Life Science Identifiers (LSIDs) can be consulted through any web browser by adding the LSID to the prefix “http://zoobank.org/ .” This publication is registered under the following LSID: urn:lsid:zoobank.org:act:9544E601‐68B5‐46C0‐B095‐0BBED2E15442.

## 3 RESULTS AND TAXONOMIC ACCOUNT

**FIGURE 2:**
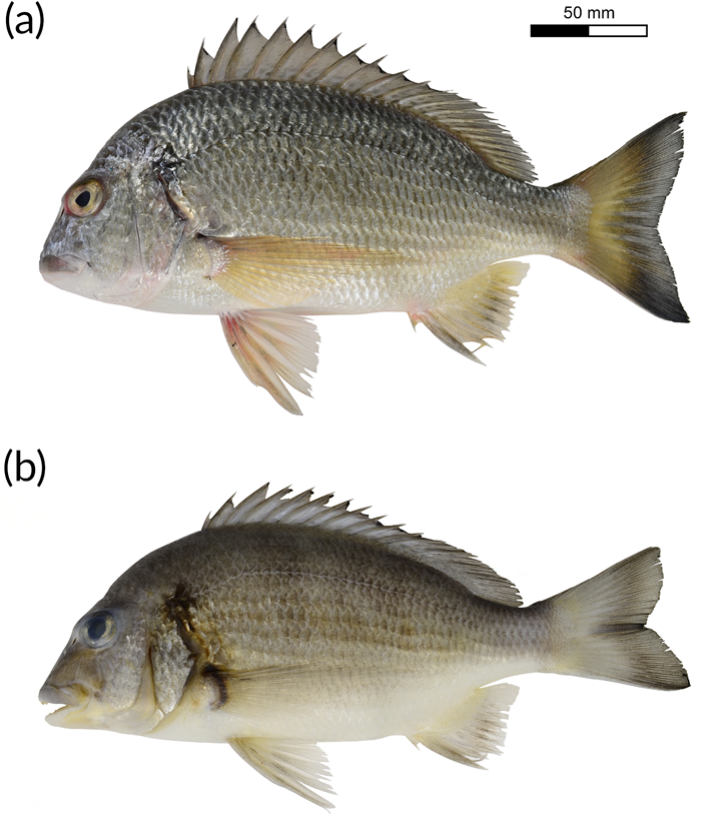
(a) Freshly collected holotype of *Acanthopagrus oconnorae* sp. nov., CAS‐ICH 247294, 222.7 mm SL (standard length), from the central Saudi Arabian Red Sea. (b) Holotype of *A. oconnorae* sp. nov. after preservation in formalin. The posterior margin of the preopercle and opercle turns darkish or blackish, and yellowish portions of pectoral, anal and pelvic fins turn hyaline after preservation. Photo credits: L. Pombo‐Ayora

**FIGURE 3:**
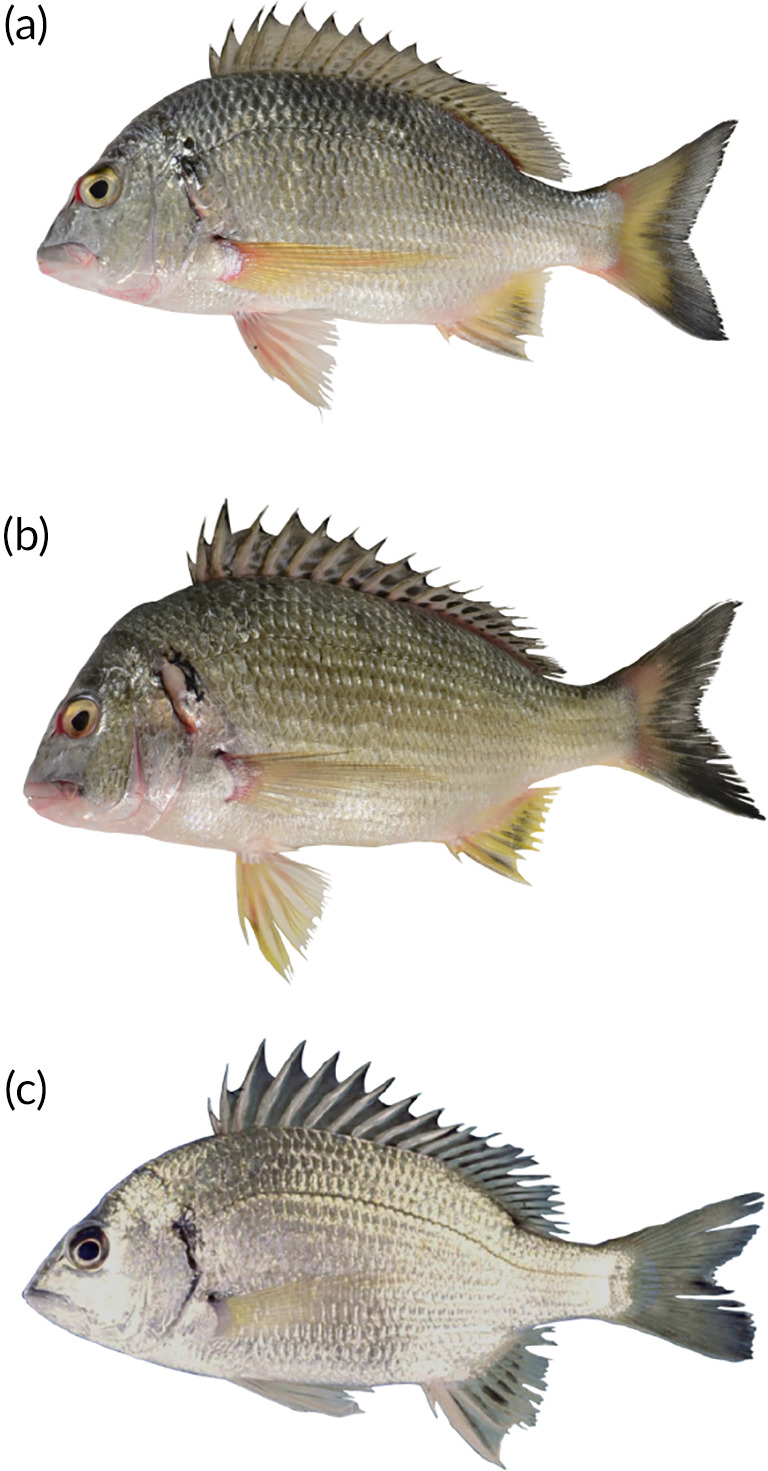
Species of *Acanthopagrus* similar to *Acanthopagrus oconnorae* currently known from the Western Indian Ocean region. (a) *A. oconnorae* sp. nov. [CAS‐ICH 247299, 185.8 mm SL (standard length), Thuwal, Red Sea]. (b) *Acanthopagrus sheim* (168.3 SL, Dammam fish market). (c) *Acanthopagrus vagus* (200 mm SL, Kosi Bay, South Africa; specimen not retained). Note the differences in the colouration of the dorsal fin and anal fin. See Table 2 for detailed morphometric comparisons. Photo credits: (a, b) L. Pombo‐Ayora, (c) Bruce Mann

*A. oconnorae* Pombo‐Ayora and Peinemann, sp. nov. (Figures 2a,b and 3a,b; Supporting Information Figures S1 and S2; Supporting Information Tables 1 and 2).

**TABLE 1:** Meristics and morphological proportions for *Acanthopagrus oconnorae* sp. nov.

	Holotype CAS‐ICH 247294	Paratypes (*n* = 10) CAS‐ICH 247295‐304
Counts		
Dorsal‐fin rays	X, 11	X, 11
Anal‐fin rays	III, 8	III, 8
Pectoral‐fin rays	15	15
Pelvic‐fin rays	I, 5	I, 5
Pored lateral‐line scales	43	42–45
Scales above lateral line	4½	4½ –5½
Scale rows between fifth dorsal‐fin ray spine base and lateral line	3½	3½
Scale rows between ninth dorsal‐fin ray spine base and lateral line	3½	3½ –4½
Gill rakers	5 + 9	5 + 9
Standard length (mm)	222.6	185.8–269.8 (221.6)
Proportions		
Body depth	42	40–45 (42)
Body depth at first anal‐fin spine origin	35	33–38 (35)
Head length	29	29–32 (30)
Body width at pectoral‐fin base	16	15–17 (16)
Snout length	12	12–14 (13)
Orbit diameter	7	7–9 (8)
Bony interorbital width	9	8–11 (9)
Upper‐jaw length	12	12–15 (13)
Caudal peduncle depth	12	12–13 (12)
Caudal peduncle length	14	12–15 (14)
Predorsal length	41	41–45 (42)
Pre‐anal length	66	64–70 (67)
Prepelvic length	35	35–39 (37)
Dorsal‐fin base	54	51–57 (54)
Anal‐fin base	16	13–17 (15)
Caudal‐fin length	22	22–28 (25)
Pelvic‐fin spine	15	13–15 (14)
First pelvic‐fin ray	21	20–24 (22)
Longest pectoral ray	33	31–36 (33)
First dorsal‐fin spine	5	5–8 (6)
Second dorsal‐fin spine	10	9–12 (11)
Third dorsal‐fin spine	14	13–15 (14)
Fourth dorsal‐fin spine	14	13–16 (15)
Fifth dorsal‐fin spine	15	14–16 (16)
Sixth dorsal‐fin spine	14	13–15 (14)
Last dorsal‐fin spine	11	10–12 (11)
First dorsal‐fin ray	11	10–13 (11)
First anal‐fin spine	4	3–5 (4)
Second anal‐fin spine	15	13–17 (15)
Third anal‐fin spine	13	12–13 (13)
First anal‐fin ray	13	10–13 (12)
Suborbital width	6	6–7 (6)
Posteriormost jaw width	13	13–15 (14)
2AS/3AS^a^	1.21	1–1.3 (1.2)
SL:BD^b^	2.4	2.2–2.5 (2.4)

*Note*: Proportion measurements are the percentages of the standard length. For paratypes, minimum and maximum are given, and the average is given in parentheses. BD, body depth; SL, standard length.

^a^ Second anal‐fin spine length/third anal‐fin spine length.

^b^ Standard length/body depth.

**TABLE 2:** Selected characteristics of *Acanthopagrus oconnorae* sp. nov. and four Western Indian Ocean congeners

	*A. occonorae* sp. nov.	*Acanthopagrus vagus* ^a^	*Acanthopagrus sheim* ^b^	*Acanthopagrus arabicus* ^b^	*Acanthopagrus berda* ^a^
Dorsal‐fin ray	XI, 11	XI–XII, 10–12	XI–XII, 11–10 (XI, 11)	XI–XII, 11–10 (XI, 11)	XI–XII, 10–12 (XI, 11)
Anal‐fin rays	III, 8	III, 8–9	III, 8 (III, 8)	III, 8 (III, 8)	III, 9 (III, 9)
Gill rakers	5 + 9 = 14	6–7 + 10–11 = 16–18	4–7 + 1 + 7–10 = 12–18 (5 + 9 = 14)	5–6 + 1 + 8–9 = 14–16 (9 + 6 = 15)	5–7 + 8–12 = 13–17 (6 + 8 = 14)
Pored lateral‐line scales	42–45	44–46	43–47 (44–46)	42–45 (46–48)	42–44 (43)
Scales above lateral line	4½	4½	4½–5½ (4½)	4½–5½ (4½)	3½–4 (4)
Scale rows between fifth dorsal‐fin ray spine base and lateral line	3½	3½	4½ (4½)	4½ (4½)	3½ (3½)
Suborbital width	6.3	4–8	4–6 (5.9–6.7)	4–6 (4.7)	2.9
Ventral edge of first to infraorbitals	Straight to weakly concave	Straight	Straight	Straight	Concave
Bulge at dorsal head profile	Absent	Absent	Absent	Absent	Absent
Strong diffuse dark blotch at origin of lateral line and in upper cleithrum and posterior upper opercle	Present	Present	Present	Present	Absent
Body colouration	Darkish silvery dorsally; gradually transitioning to silvery ventrally	Bluish silver with iridescent scales dorsally, ventral colour pale to whitish with somewhat yellowish silvery reflections	Head and body silvery pale grey, belly whitish silver, weak streaks along longitudinal scale rows	Head and body silvery pale grey, belly whitish; very weak silver streaks along longitudinal rows of scales	Dull dark olive brown above, pale to whitish, with dark silver brassy reflections below
Dorsal‐fin colouration	Overall grey; subtle light‐yellow colouration near posterior margin; some irregular dark blotches on inter‐radial membranes at base	Spinous dorsal fin bluish grey anteriorly, margin pale yellowish	Dorsal fin greyish to hyaline or blackish grey, fin membrane similar but with dark spots	Dorsal fin grey to hyaline or blackish grey	Dorsal fin dusky, membrane of spinous dorsal fin darker, a few, somewhat longitudinal, bands on membranes of soft rays
Anal‐fin colouration	Grey to yellowish grey anteriorly; yellow or yellowish basally and posteriorly; no streaks on inter‐radial membranes	Yellow or transparent with black streaks on inter‐radial membranes	Black streaks proximally on inter‐radial membranes between anal‐fin rays	Yellow	Uniformly grey
Caudal‐fin colouration	Light yellow with broad black posterior margin (approximately half of caudal fin)	Caudal fin brownish at the centre, dark grey to black distally and along dorsal and ventral margins	Blackish grey, lower margin of caudal fin yellow	Blackish grey, lower margin of caudal fin bright yellow	Caudal‐fin membrane slightly darker brown than body, posterior margin often darker

*Note*: Values in parentheses correspond to the individuals that were analysed in this study.

^a^ Iwatsuki and Heemstra (2010).

^b^ Iwatsuki (2013).

### 3.1 Holotype

CAS‐ICH 247294, 222.7 mm SL (standard length), Thuwal, Saudi Arabia, 22.308367° N, 39.091016° E, coll. C. Williams, L. Pombo‐Ayora and V. Peinemann, 24 August 2021.

### 3.2 Paratypes

CAS‐ICH 247295, 190.0 mm SL, Thuwal, Saudi Arabia, 22.339688° N, 39.092005° E, coll. D. Bradley, 30 July 2021. CAS‐ICH 247296‐247297, two specimens, 218.7–244.0 mm SL, Thuwal, Saudi Arabia, 22.307979° N, 39.091454° E, coll. C. Williams, 25 August 2021. CAS‐ICH 247298, 237.5 mm SL, Thuwal, Saudi Arabia, 22.308367° N, 39.091016° E, coll. C. Williams, L. Pombo‐Ayora and V. Peinemann, 23 August 2021. CAS‐ICH 247299‐247300, two specimens 185.4–239.6 mm SL, Thuwal, Saudi Arabia, 22.308367° N, 39.091016° E, coll. C. Williams, L. Pombo‐Ayora and V. Peinemann, 24 August 2021. CAS‐ICH 247301‐247304, four specimens 200.3–269.8 mm SL, Thuwal, Saudi Arabia, 22.308367° N, 39.091016° E, coll. S. He, L. Pombo‐Ayora and V. Peinemann, 26 August 2021.

### 3.3 Diagnosis

**FIGURE 4:**
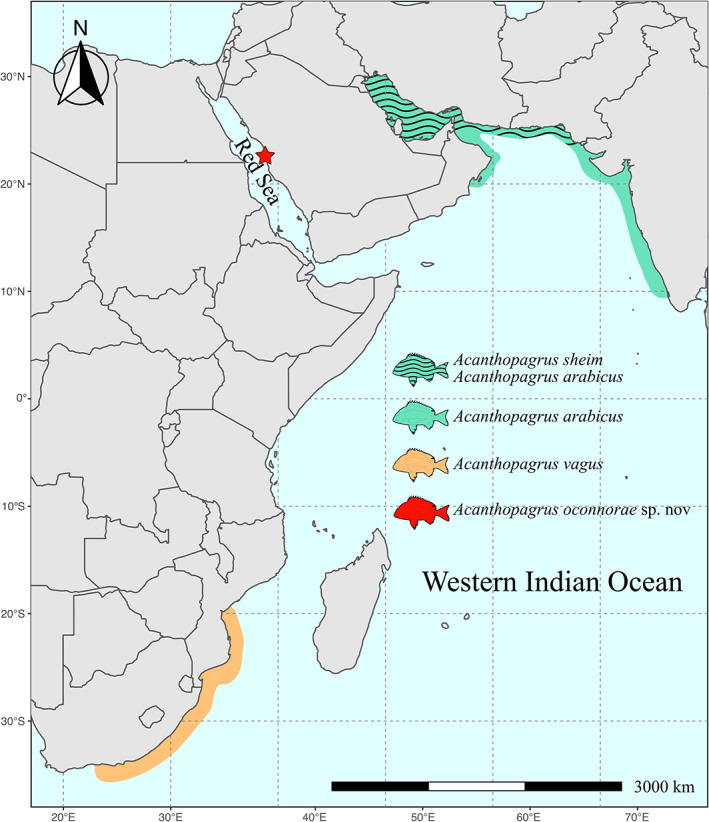
Map illustrating the type locality of *Acanthopagrus oconnorae* sp. nov. (red star) and the geographical distribution of *Acanthopagrus arabicus* (green and dashed green), *Acanthopagrus sheim* (dashed green) and *Acanthopagrus vagus* (orange)

*A. oconnorae* is distinguished from its congeners by the following set of characters: dorsal fin XI, 11; anal fin III, 8; 4½ scale rows above lateral line; 3½ scale rows between fifth dorsal‐fin spine and lateral line; suborbital width 6%–7% of SL; body moderately deep (40%–45% of SL); head length 29%–32% of SL; second anal‐fin spine 13%–17% of SL; anal fin yellowish grey or dusky grey, with posterior one‐fifth of the fin light yellow; black streaks on inter‐radial membranes of anal fin absent; caudal fin light yellow with a broad black posterior margin (approximately half of the fin); vertical bands on body absent or weak (four horizontal scale rows wide, if present); conspicuous black spot on the upper base of pectoral fin; diffuse black blotch at the origin of lateral line covering the upper part of the cleithrum (Figure 4).

### 3.4 Description

**FIGURE 5:**
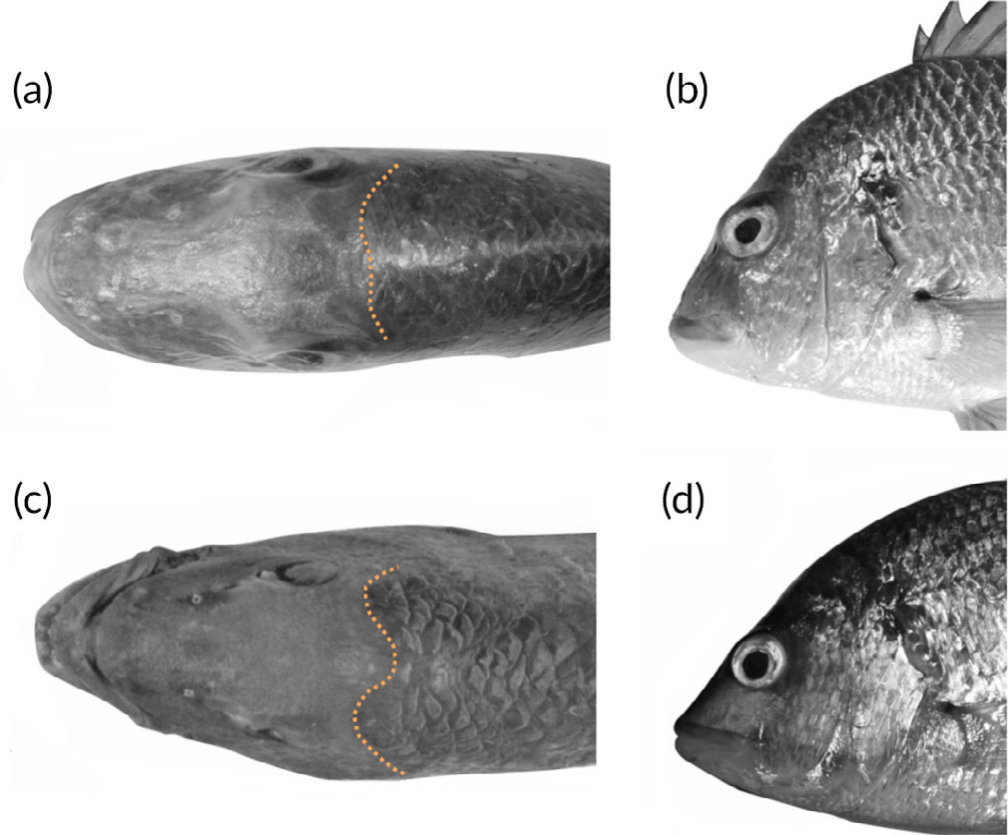
Left: scale patterns of the anterior edge of the scaled predorsal area. Right: lateral head profile. (a, b) *Acanthopagrus oconnorae*, (c, d) *Acanthopagrus vagus*. Photo credits: (a, b) L. Pombo‐Ayora, (c, d) modified from Iwatsuki and Heemstra (2010)

Counts and measurements are presented in Table 1. Counts and measurements of individual paratypes are presented in Supporting Information Table S2, and photos of type specimens are shown to scale in Supporting Information Figure S1. Body compressed and moderately deep; mouth horizontal, maxilla reaching to below middle of pupil; ventral edge of first two infraorbitals straight to weakly concave; two nostrils in front of each eye, posterior nostril slit‐like, anterior nostril rounded and in line with ventral margin of the orbit; orbit diameter always subequal to bony interorbital width; eyes positioned at anterior edge of head, often forming a weakly convex break in an otherwise gently curved head profile, when viewed laterally (see Figure 5b); teeth in upper and lower jaws (generally four rows and three rows, respectively), smaller and irregular rows anteriorly, typical molariform teeth strongly developed posteriorly; usually six canine‐like teeth anteriorly in upper jaw (but sometimes absent leaving a gap), six in lower jaw; suborbital width subequal to orbit diameter; anterior edge of scaled predorsal area curved (Figure 5); fourth or fifth dorsal‐fin spine longest (1 of 11 specimens with sixth dorsal‐fin spine longest); first anal‐fin spine short, clearly less than orbit diameter; second anal‐fin spine slightly longer than the third (2AS/3AS 1.0–1.3); pectoral‐fin tip reaching to level with second or third anal‐fin spine.

### 3.5 Colour in life

Based on the holotype (Figure 2a) and paratypes (Supporting Information Figure S1): head and body silvery grey; body gradually transitions from dark silvery grey dorsally and posteriorly to a white silvery grey ventrally; upper lip grey; lower lip white; dorsal fin grey, some specimens with subtle light yellow colouration near posterior margin; most specimens with some irregular dark blotches on inter‐radial membranes of dorsal‐fin base; caudal fin light yellow with a broad black posterior margin (approximately half of caudal fin); pelvic fin whitish, light red anteriorly; pectoral fin yellowish hyaline, a conspicuous black spot on the upper base; anal fin dusky grey, with posterior one‐fifth of fin yellow (primarily light yellow from sixth to eighth anal‐fin ray); a diffuse black blotch at the origin of the lateral line covering the upper part of the cleithrum; vertical bands on body absent or weak (four horizontal scale rows wide, if present); diffuse, dark grey horizontal band between eyes evident in live individuals.

### 3.6 Colour in preservative

Figure 2(b) shows the holotype colouration after fixation in formalin: head and body silvery grey; body transitions to white ventrally, completely white below the ventral margin of the pectoral‐fin base; upper lip grey; anterior portion of upper lip white ventrally; lower lip white; dorsal fin grey; most specimens with some irregular dark blotches on inter‐radial membranes of the dorsal‐fin base; caudal fin light grey, posterior half darker grey in most specimens; pelvic fins clear white; pectoral fins transparent with a dark grey to black base; anal fin dusky grey with transparent membrane distally and posteriorly; a diffuse black blotch at the origin of the lateral line covering the upper part of the cleithrum; vertical bands on body absent or weak (four horizontal scale rows wide, if present).

### 3.7 Distribution and habitat

Currently this species is known from the mangrove‐adjacent sandflats and mangrove‐encircled pools of Thuwal, Saudi Arabia, in the central Red Sea. All specimens were caught in very close proximity to the mangrove habitat. All the trapped specimens were captured on sandflat shelves with very shallow water (maximum 1 m depth at high tide) near coastal stands of mangroves (*Avicennia marina*). Individuals of *A. oconnorae* appear to commonly utilize a specific type of habitat, co‐occurring with *A. berda*, *R. haffara*, *Pomadasys argenteus*, *Gerres longirostris*, *Monodactylus argenteus*, *Albula glossodonta* and *Crenimugil crenilabis*.

### 3.8 Etymology

*A. oconnorae* is named in honour of Winefride Bradley (née O'Connor), botanist, on the occasion of her 90th birthday. D.D.C.B., her son, first noted several of the distinctive features of this fish in specimens caught while leisure fishing, and he provided a caudal‐fin clipping for initial genetic analysis. D.D.C.B. collected the first specimen (CAS‐ICH 247295) analysed in this study.

### 3.9 Common name

The following common name is proposed: Bev Bradley's Bream, after D.D.C.B.'s wife, Mrs. Beverley Bradley.

### 3.10 Remarks

Despite frequent visits to local fish markets (*e.g*., Shellum *et al*., 2021), none of the authors have seen *A. oconnorae* at any fish market. This may suggest that it is restricted to habitats that receive relatively little fishing pressure, such as the shallow mangrove sandflats and mangrove‐encircled pools in which the type specimens were collected. Although it is so far confirmed to occur only in Thuwal, *A. oconnorae* is potentially more widely distributed throughout the Red Sea in mangrove and sandflat habitats. *A. berda*, which co‐occurs with *A. oconnorae* in Thuwal's mangrove habitats, is reported to occur in the entire Red Sea from the Gulf of Aqaba (Ben‐Tuvia & Steinitz, 1952) to Yemen (Forsskål, 1775).

### 3.11 Comparisons

Selected characteristics highlighting the morphological taxonomy differences between *A. oconnorae* sp. nov. and other similar species in the Western Indian Ocean region are summarized in Table 2. The three species most similar to *A. oconnorae* are illustrated in Figure 3 along with their geographical distribution in Figure 4. *Acanthopagrus* species with similar colouration and Western Indian Ocean distribution are shown in Supporting Information Figure S2.

*A. oconnorae* shares somewhat similar fin colouration with *A. arabicus*, *A. sheim* and *A. vagus*. All have more or less yellow or yellowish pelvic fin and anal fin. Of these, *A. oconnorae* is the most similar to *A. vagus* from the south‐western Indian Ocean (Table 2). Both have 3½ scale rows between the fifth dorsal‐fin spine base and lateral line. Nonetheless, they differ in the number of gill rakers (14 in *A. oconnorae* compared to 16–18 in *A. vagus*) and the colouration of fins; the anal fin of *A. oconnorae* is yellowish grey or dusky grey with a yellow basal and posterior margin, whereas *A. vagus* exhibits pale yellow anal fins with black streaks near the base on each inter‐radial membrane (Figure 3b) (Iwatsuki & Heemstra, 2010). *A. oconnorae* can additionally possess irregular dark blotches on the inter‐radial membranes of the dorsal‐fin base (Supporting Information Figure S1), which are not present in *A. vagus*. Moreover, *A. oconnorae* has a relatively blunt snout and an almost curved scaleless area anterior region of the nape, whereas *A. vagus* has a more pointed snout and a w‐shaped anterior nape scaleless region (Iwatsuki & Heemstra, 2010) (Figure 5). Head profile comparisons between *A. oconnorae* and *A. vagus* are based on specimens shown in Iwatsuki and Heemstra (2010), as well as four specimens of *A. vagus* deposited at the South African Institute of Aquatic Biodiversity. Those individuals, under the records ACEP 09‐308, ACEP09‐328, ACEP09‐329 and ACEP09‐330 in the Global Biodiversity Information Facility, were identified as *A. berda* in 2009. Nonetheless, they clearly match the description of *A. vagus*, in that they have black streaks on the inter‐radial membranes of the anal fin and no concavity at the ventral edge of the first two infraorbitals (Iwatsuki & Heemstra, 2010).

*A. oconnorae* differs from both *A. arabicus* and *A. sheim* in the count of scale rows between the fifth dorsal‐fin spine base and lateral line (3½ *vs*. 4½) and in fin colouration (see Table 2). *A. arabicus* has bright yellow pelvic, anal and ventral edges of the caudal fin and no black streaks proximally on inter‐radial membranes between yellow anal‐fin rays, and *A. sheim* has pelvic fins usually whitish with pinkish tinge, anal fin mostly yellow with black markings in the middle, ventral edge of caudal fin usually orange pink or rarely yellowish, with black streaks present on inter‐radial membranes of fin rays (Iwatsuki, 2013; Sergey Bogorodsky, pers. comm.). Nonetheless, two or three series of small black blotches are present on the proximal inter‐radial membranes between the dorsal‐fin rays in *A. sheim*, whereas *A. oconnorae* has small, irregular blotches infrequently (Supporting Information Figure S1).

*A. oconnorae* differs from *A. omanensis* and *A. randalli* in the number of scale rows between the fifth dorsal‐fin spine base and lateral line (3½ *vs*. 5½ *vs*. 4½) and in body colouration. *A. omanensis* has distinctive broad black margins in its dorsal, caudal, anal and pelvic fins, which contrast with its bright silvery body (Iwatsuki & Heemstra, 2010). *A. randalli*, an Arabian Gulf endemic, typically has four to five broad (six to seven scale horizontal row width) vertical bands across its body, whereas *A. oconnorae*, if present at all, has four to five faint narrow bands (four scale horizontal row width). Furthermore, *A. oconnorae* lacks the characteristic eye bulge present in *A. randalli* (Iwatsuki & Carpenter, 2009).

*A. oconnorae* is presumably a Red Sea endemic. It is easily distinguishable from the two other currently recognized species of *Acanthopagrus* reported from the Red Sea (*A. berda* and *A. bifasciatus*). *A. berda* has the same number (3½) of scale rows between the fifth dorsal‐fin spine base and lateral line as *A. oconnorae*, but it has a much deeper body, uniformly dark grey to brown body and median fins, and a distinctive concavity at the ventral edge of the first two infraorbitals (Iwatsuki & Heemstra, 2010). *A. bifasciatus* and *A. catenula* are quite dissimilar to *A. oconnorae* by their conspicuous black bars across the faces and black pelvic and anal fins and bright yellow dorsal, pectoral and caudal fins (Iwatsuki & Heemstra, 2011). Moreover, they have 5½–6½ and 4½ scale rows, respectively, between the fifth dorsal‐fin spine base and lateral line (Iwatsuki & Heemstra, 2011), whereas *A. oconnorae* has 3½ scale rows.

### 3.12 Phylogenetic analysis

**FIGURE 6:**
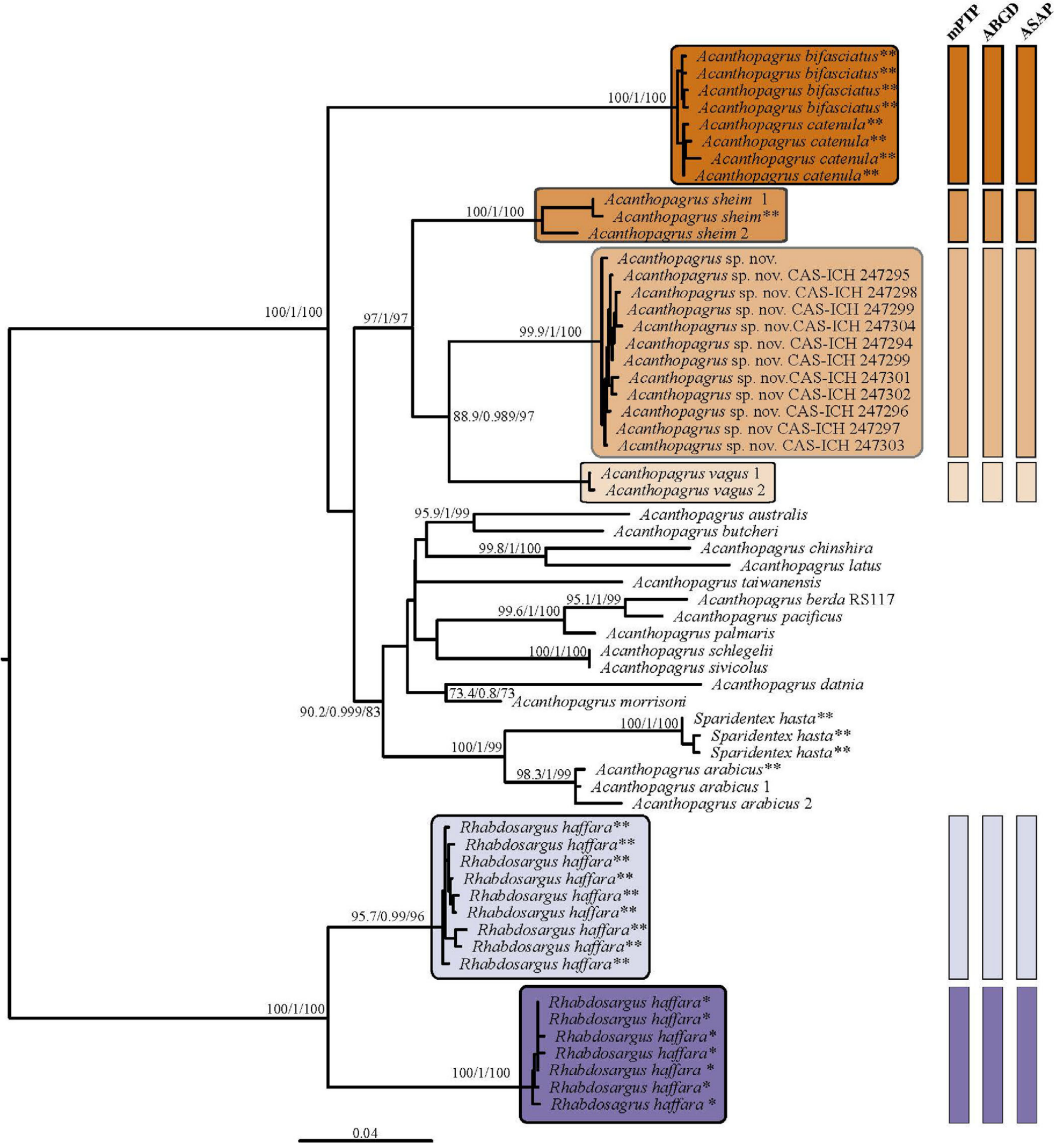
Maximum likelihood phylogenetic tree of the concatenated alignment of the mitochondrial regions COI, CytB and 16S. Posterior probability derived from SH‐aLRT support (%), approximate Bayes test and ultrafast bootstrap. Support metrics for the nodes with values <80, <0.95 and <95, respectively, are not shown. *Rhabdosargus haffara* was used as an out‐group. Coloured panels on the right indicate species delimitations obtained from mPTP (multi‐rate Poisson tree process), ABGD (Automatic Barcode Gap Discovery) and ASAP (Assemble Species by Automatic Partition). Species with asterisks correspond to samples obtained either from the Red Sea (*) or the Arabian Gulf (**). Note that a fin clip from one individual of *Acanthopagrus oconnorae* was sequenced, but the specimen was not retained; thus, there is no catalogue number for one individual in the tree

Here the authors present the most comprehensive molecular phylogeny of the genus *Acanthopagrus* to date, including 17 of the 20 currently described species and the new species described here (*A. oconnorae*) (Figure 6; Appendix 1). The maximum likelihood tree supported by Bayesian posteriors of 1 indicates that all the specimens the authors collected of *A. oconnorae* form a monophyletic group and that its closest relative (Bayesian posterior support 0.98) is *A. vagus*, whose geographic distribution does not include the Red Sea (it is present in the Indian Ocean from South Africa to southern Mozambique, Figure 4) (Iwatsuki & Heemstra, 2010). With Bayesian posterior support of 1, the closest species to the clade composed of *A. oconnorae* and *A. vagus* is *A. sheim*, which is known only from Pakistan and the Arabian Gulf. These results were supported by the K2P distances of COI and 16S (Table 3). The COI matrix indicates that the closest species to *A. oconnorae* is *A. vagus* with a mean distance of 7.2% followed by *A. sheim* with a mean distance of 7.9% and *A. arabicus* with a mean distance of 9.4% (Table 2). As noted by previous authors (Carpenter & Johnson, 2002; Hasan *et al*., 2020), *S. hasta* is placed in the genus *Acanthopagrus* as a monophyletic group with *A. arabicus* (Bayesian posterior support 1). Further consideration and possible revision of the genus *Acanthopagrus* and careful examination of *Sparidentex* may be necessary for future studies, but it is beyond the scope of the present work.

**TABLE 3:** Interspecific genetic divergences of *Acanthopagrus* spp. from the Western Indian Ocean

COI	*n*	*Acanthopagrus arabicus*	*Acanthopagrus sheim*	*Acanthopagrus oconnorae* sp. nov.	*Acanthopagrus vagus*	*Acanthopagrus berda*	*Acanthopagrus bifasciatus*	*Acanthopagrus catenula*
*A. arabicus*	12	–	0.0116	0.0129	0.0123	0.0131	0.0139	0.0136
*A. sheim*	16	0.0858	–	0.0115	0.0108	0.0123	0.0144	0.0140
*A. oconnorae* sp. nov.	12	0.0943	0.0792	–	0.0115	0.0139	0.0156	0.0153
*A. vagus*	4	0.0903	0.0778	0.0725	–	0.0133	0.0152	0.0149
*A. berda*	3	0.1009	0.0919	0.1015	0.0898	–	0.0146	0.0143
*A. bifasciatus*	4	0.1146	0.1117	0.1181	0.1149	0.1158	–	0.0031
*A. catenula*	4	0.1150	0.1105	0.1178	0.1155	0.1149	0.0086	–
16S								
*A. arabicus*	9	–	0.0084	0.0095	NA	0.0090	0.0110	0.0109
*A. sheim*	15	0.0387	–	0.0078	NA	0.0090	0.0124	0.0124
*A. oconnorae* sp. nov.	12	0.0449	0.0328	–	NA	0.0094	0.0098	0.0096
*A. vagus*	–	NA	NA	NA	–	NA	NA	NA
*A. berda*	3	0.0464	0.0441	0.0464	NA	–	0.0121	0.0122
*A. bifasciatus*	4	0.0609	0.0708	0.0469	NA	0.0699	–	0.0012
*A. catenula*	3	0.0595	0.0701	0.0446	NA	0.0703	0.0022	–

*Note*: The genetic divergence is estimated by K2P distances calculated based on COI sequences (upper table) and 16S sequences (lower table). For each marker's section, the K2P distance values are presented in the lower matrix, whereas the corresponding standard deviation of each value is shown in the upper matrix. NA, not available.

All molecular species delimitation methods used indicate that *A. oconnorae* is a genetically distinct species from its congeners (at least for the 17 species that have sequences deposited in GenBank). Interestingly, these analyses revealed further unresolved species inside the genus *Acanthopagrus*. Samples of *A. bifasciatus* from the Arabian Gulf and samples of *A. catenula* from Oman are clustered together and appear as a single genetic clade; this pattern was revealed by the mPTP analysis (which uses the mitochondrial phylogeny) and by the ABGD and ASAP methods (based on COI alignments). The K2P divergence distances were minimal between *A. bifasciatus* and *A. catenula*, with 0.8% and 0.2% differences for COI and 16S fragments, respectively (Table 3).

On the contrary, the out‐group composed of samples of *R. haffara* from the Red Sea and the Arabian Gulf appears as genetically separated species differentiated by their geographical origin. A full morphological comparison of the individuals of *R. haffara* from the Arabian Gulf and the Red Sea is warranted to further investigate if these should be considered two distinct species.

## 4 DISCUSSION

The Red Sea is a biodiversity hotspot (Dibattista *et al*., 2016a, 2016b), with one of the highest levels of endemism in the world. With 14.6% of Red Sea fish species found nowhere else, the Red Sea is behind only the Hawaiian Islands (30.4%) and Easter Island (21.7%) in its proportion of endemism for fishes (Bogorodsky & Randall, 2018). Such a high biodiversity and endemism rate appear to be firmly linked with the unique environmental characteristics (*e.g*., high temperatures and salinity) found in the Red Sea (Berumen *et al*., 2019). Due to its status as a relatively understudied locality (Berumen *et al*., 2013), the potential for the discovery of new Red Sea species remains high. In some cases, careful examination of widespread taxa has revealed cryptic diversity (*e.g*., Coleman *et al*., 2016; Priest *et al*., 2016), indicating that the level of endemism in the Red Sea may be underestimated. As a relatively large (maximum 27 cm total length) sparid fish inhabiting shallow coastal waters in close proximity to a large human population, the newly described *A. oconnorae* exemplifies this potential.

Endemic lineages in the Red Sea are, in most cases, derived from their Indian Ocean counterparts (DiBattista *et al*., 2013, 2015), potentially resulting from allopatric divergence during Pleistocene isolation (Klausewitz, 1989). *A. oconnorae* appears to be a sister species to *A. vagus* from the south‐western Indian Ocean. *A. oconnorae* is considerably distinct from *A. vagus*, both morphologically and genetically (7.2% K2P COI divergence), unlike *Crenidens crenidens*, another sparid, which shows relatively low divergence and very low morphometric variation between the two lineages of the Red Sea and the south‐western Indian Ocean (Bogorodsky *et al*., 2017).

The behaviour and habitat of *A. oconnorae* may provide some explanation regarding why this species remained undetected for so long, especially considering that early natural historians (including Forsskål, Linnaeus, Ehrenberg and Rüppell) began describing Red Sea fauna more than 200 years ago, frequently using material from local fishermen (see Berumen *et al*., 2019). Modern local fishing operations still focus on reef fish, employing hand‐lines, nets, traps and other methods (Jin *et al*., 2012; Tesfamichael & Pauly, 2016), and these are mostly utilized in the slightly deeper waters of the fringing reefs or further away from the coastline. Therefore, very little fishing effort is concentrated in the very shallow waters immediately adjacent to or encircled by mangrove habitats. A potential hypothesis to explain the prior lack of detection of *A. oconnorae* is that the species' behaviour is to remain in very close proximity to the mangroves, whereas species such as *A. berda* venture out into the deeper waters of the fringing reef (Garratt, 1993), where they are routinely captured in the local fisheries (notably, the holotype of *A. berda* was collected by Forsskål between 1761 and 1763 during the Danish Arabian expedition). Further work on the fine‐scale distribution, habitat use and movement ecology of *A. oconnorae* could test this hypothesis.

The discovery of a relatively large species in such proximity to inhabited areas further underscores the need for immediate conservation actions. Several large coastal developments are underway in Saudi Arabia; care must be taken to avoid the degradation of habitats such as the mangrove and sandflats that *A. oconnorae* appears to utilize. Such habitat specialization is likely to occur with numerous less‐conspicuous species (*e.g*., Atta *et al*., 2019; Troyer *et al*., 2018); there are potentially many additional undescribed species in the Red Sea remaining to be discovered in families such as the Gobiidae (*e.g*., Coker *et al*., 2018). The geographic range of *A. oconnorae* remains unknown, but it is likely that it could occupy similar habitats throughout the Red Sea. At present, it should be considered a Red Sea endemic. Extensive baseline surveys can help address such issues, especially in unique habitats and in areas where development is planned.

## AUTHOR CONTRIBUTIONS

D.D.C.B., L.P.‐A., V.N.P., S.H., Y.J.L. and C.T.W. collected samples; L.P.‐A., V.N.P. and C.T.W. produced DNA sequences; L.P.‐A., V.N.P. and S.H. analysed the molecular data; L.P.‐A., V.N.P., Y.I. and Y.J.L. analysed the morphological data; L.P.‐A. and V.N.P. described the new species; L.P.‐A. and V.N.P. led the first draft of the manuscript. All authors made significant contributions to the manuscript.

## Supporting information


**FIGURE S1** Freshly collected type specimens of *Acanthopagrus oconnorae* sp. nov., shown to scale. Labels indicate catalogue numbers at the California Academy of Sciences. Photo credits: L. Pombo‐Ayora

**FIGURE S2** Species of *Acanthopagrus* with similar colouration to *Acanthopagrus oconnorae* with Western Indian Ocean distribution. (a, b) *A. oconnorae* sp. nov. (CAS‐ICH 247304, 269.8 mm SL, and CAS‐ICH 247299, 185.8 mm SL). (c) *A. berda*. (d) *A. sheim*. (e) *A. arabicus*. (f) *A. vagus*. Photo credits: (a–e) L. Pombo‐Ayora, (f) Bruce Mann

**TABLE S1** Collection details of all specimens examined in this study. This list clarifies the source [field collection, fish market or GenBank (an online genetic database)] for each specimen. Field collections were conducted in the Saudi Arabian Red Sea near Thuwal. All specimens obtained in a fish market in Dammam are assumed to be from the Arabian Gulf, Saudi Arabia, except for specimens of *Acanthopagrus catenula*, which are presumably imported from Oman. “GenBank” indicates that genetic sequences were obtained from an online database and a physical specimen was not examined. The column “Utilization” indicates if the specimen was used for morphological comparison, molecular study or both. The columns COI, CytB and 16S reflect the GenBank accession number for each genetic marker, where applicable. Care was taken to select sequences from reliable sources to avoid potential problems introduced by mislabelled species. The accession number can be used to cross‐reference in GenBank for information on authors and collection locality for each associated sequence. For six specimens, numbers following the species ID correspond to individuals numbered accordingly in the phylogenetic tree in Figure 6

**TABLE S2** Meristics and morphometric measurements of all *Acanthopagrus oconnorae* type specimens are described in this study. Morphometric measurements are shown as the percentage of standard length. The header row (first row) indicates the catalogue number (registered at the California Academy of Sciences) for each individual studied. All measurements were made following Hubbs and Lagler (1964)
**Appendix file 1** Concatenated alignment of the CO1, CytB and 16S data for all sparid species used in the phylogenetic analyses. Data are provided in fasta format
