# Supplementary figures and images for: Acanthopagrus oconnorae, a new species of seabream (Sparidae) from the Red Sea
Source: J Fish Biol. 2022 Aug 5;101(4):885–97. doi: 10.1111/jfb.15147 (PMC9805087; doi:10.1111/jfb.15147)

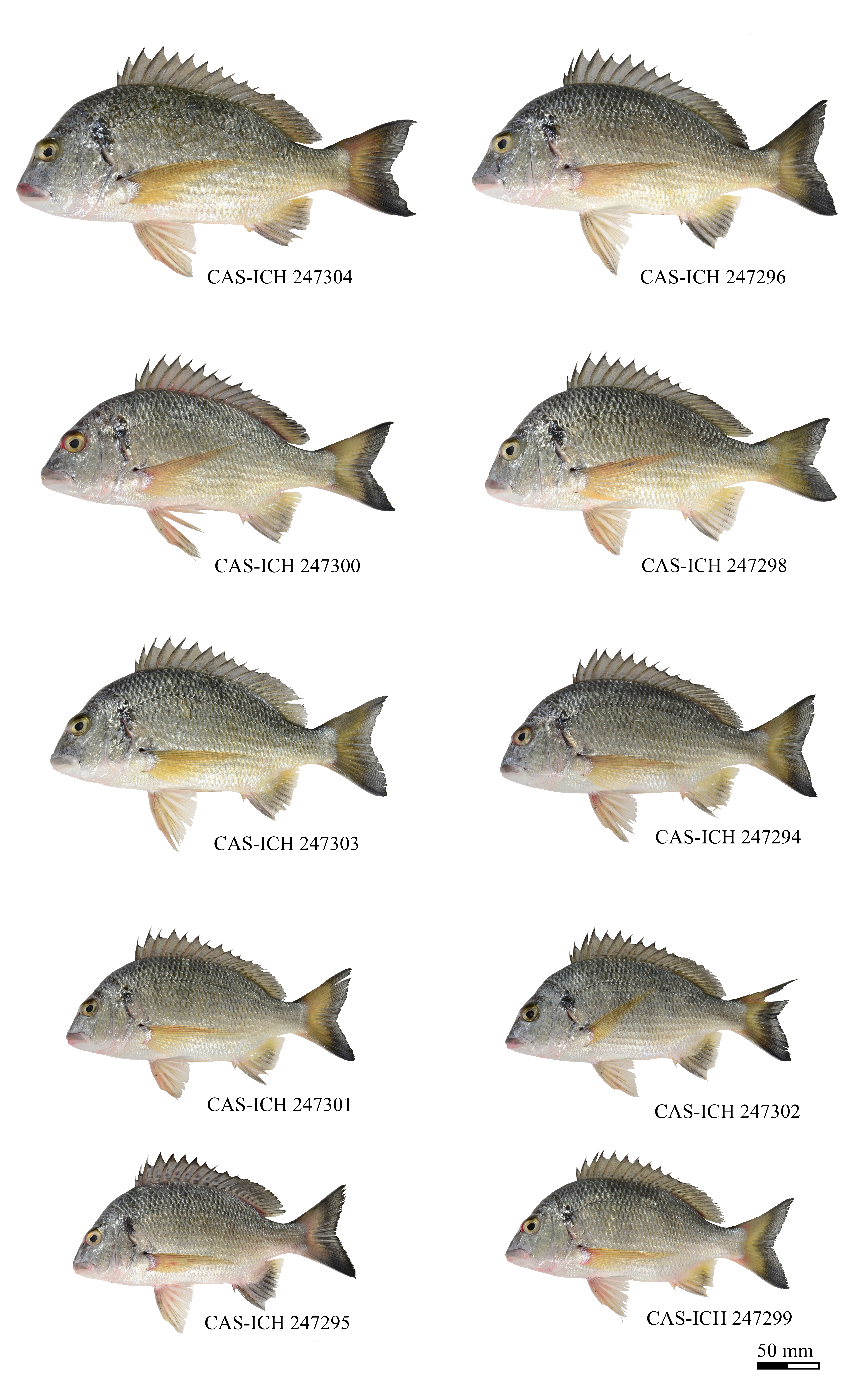

Supplement: Supplementary file 1 — FIGURE S1 Freshly collected type specimens of Acanthopagrus oconnorae sp. nov., shown to scale. Labels indicate catalogue numbers at the California Academy of Sciences. Photo credits: L. Pombo‐Ayora [file JFB-101-885-s002.png]

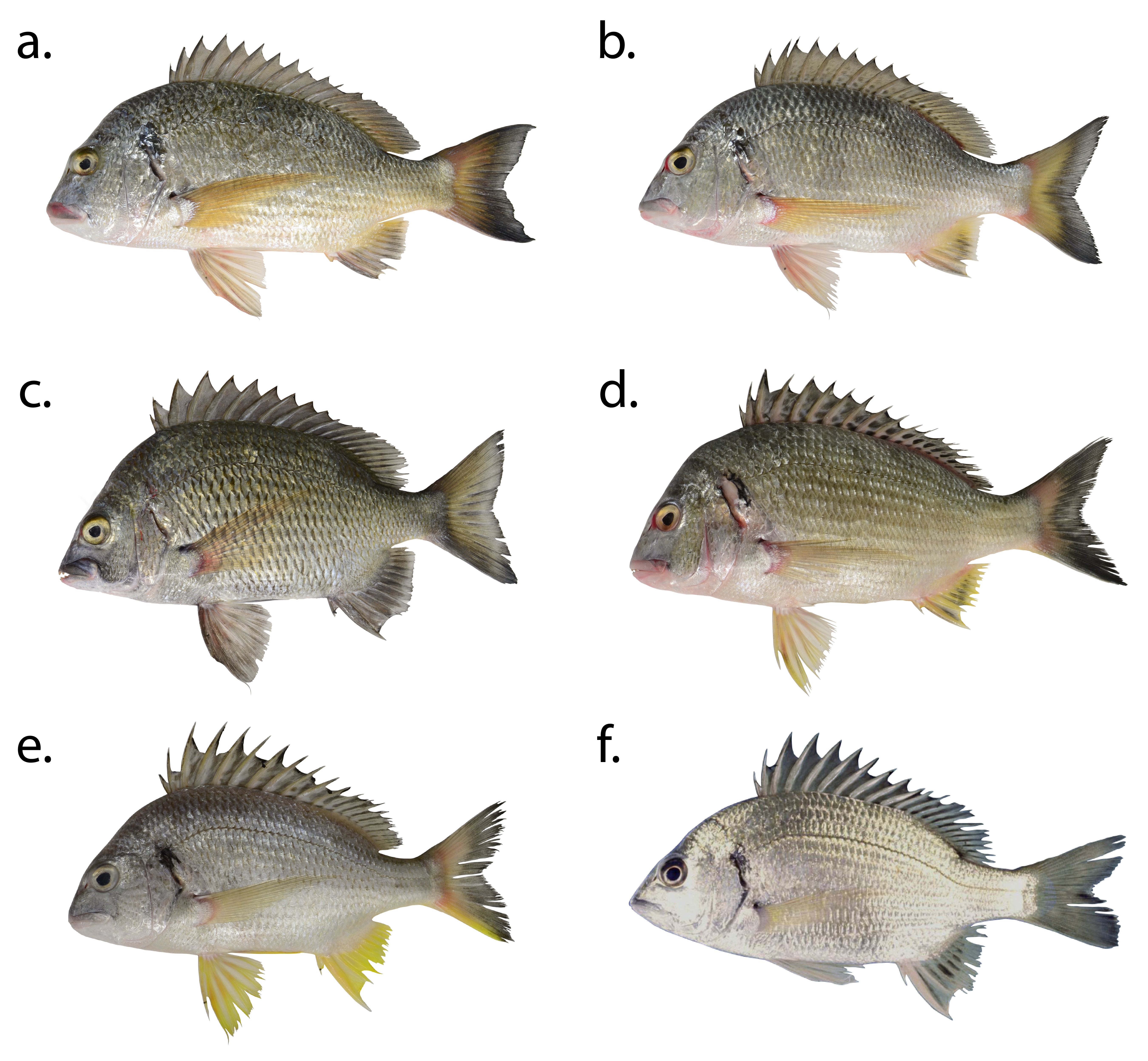

Supplement: Supplementary file 2 — FIGURE S2 Species of Acanthopagrus with similar colouration to Acanthopagrus oconnorae with Western Indian Ocean distribution. (a, b) A. oconnorae sp. nov. (CAS‐ICH 247304, 269.8 mm SL, and CAS‐ICH 247299, 185.8 mm SL). (c) A. berda. (d) A. sheim. (e) A. arabicus. (f) A. vagus. Photo credits: (a–e) L. Pombo‐Ayora, (f) Bruce Mann [file JFB-101-885-s001.png]
